# Blood Pressure Indices and Associated Risk Factors in a Rural West African Adult Population: Insights from an AWI-Gen Substudy in Ghana

**DOI:** 10.1155/2020/4549031

**Published:** 2020-04-26

**Authors:** Godfred Agongo, Engelbert A. Nonterah, Lucas Amenga-Etego, Cornelius Debpuur, Michael B. Kaburise, Stuart A. Ali, Nigel J. Crowther, Michèle Ramsay, Abraham R. Oduro

**Affiliations:** ^1^Navrongo Health Research Centre, Navrongo, Ghana; ^2^Sydney Brenner Institute for Molecular Bioscience, Faculty of Health Sciences, University of the Witwatersrand, Johannesburg, South Africa; ^3^Division of Human Genetics, National Health Laboratory Service and School of Pathology, Faculty of Health Sciences, University of the Witwatersrand, Johannesburg, South Africa; ^4^Julius Global Health, Julius Center for Health Sciences and Primary Care, University Medical Centre Utrecht, Utrecht University, Utrecht, Netherlands; ^5^West African Centre for Cell Biology of Infectious Pathogens, Department of Biochemistry, Cell and Molecular Biology, University of Ghana, Accra, Ghana; ^6^Department of Chemical Pathology, National Health Laboratory Service and School of Pathology, Faculty of Health Sciences, University of the Witwatersrand, Johannesburg, South Africa

## Abstract

Systolic (SBP) and diastolic blood pressure (DBP) are commonly used for cardiovascular disease (CVD) risk prediction, and pulse pressure (PP) and mean arterial blood pressure (MAP) can provide additional information. It is therefore important to understand the factors associated with these cardiovascular risk markers. This cross-sectional study involved 1839 men and women aged 40–60 years. Data on SBP, DBP, MAP, PP, sociodemography, lifestyle, anthropometry, and lipids were collected. Gender-stratified linear regression analyses were performed to determine the association between log-transformed blood pressure indices and the study variables. Age was associated with all measured blood pressure indices (*p* < 0.001) among men and women. Men had higher SBP (*p*=0.007) and PP (*p* < 0.001) than women. Nankana ethnicity was associated with higher PP levels (*p* < 0.005) in the total population. Vendor meal consumption among women was associated with higher PP levels (*p* < 0.05). Fruit intake among men was associated with lower PP levels (*p* < 0.05). Currently unmarried women had higher SBP (*p* < 0.005), DBP (*p* < 0.05), MAP (*p* < 0.005), and PP (*p* < 0.005) than currently married women. Pesticide exposure was negatively associated with SBP (*p* < 0.005), DBP (*p* < 0.005), MAP (*p* < 0.005), and PP (*p* < 0.05) among women. Increased subcutaneous fat was associated with DBP (*p* < 0.005) and MAP (*p* < 0.05) among women. Among men, hip circumference was associated with higher DBP and MAP (*p* < 0.05 for both associations), subcutaneous fat associated with higher SBP (*p* < 0.005), DBP (*p* < 0.001), and MAP (*p* < 0.001) and visceral fat was associated with higher PP (*p* < 0.05). In the total population, visceral fat was associated with higher DBP (*p* < 0.05) and MAP (*p* < 0.001). High-density lipoprotein cholesterol was positively associated with SBP (*p* < 0.005), DBP (*p* < 0.005), and MAP (*p* < 0.001) for women and positively associated with SBP, DBP, and MAP (*p* < 0.001 for all three) and PP (*p* < 0.05) for men. The association of blood pressure indices with modifiable risk factors suggests that targeted health interventions may reduce CVD risk in this population.

## 1. Introduction

Though the prevalence of cardiovascular diseases (CVDs) is greater in high-income than low- and middle-income countries (LMIC), the rate of premature CVD-related deaths is higher in LMIC [1]. One of the key CVD risk factors is hypertension, which is estimated to account for nearly 10.4 million deaths worldwide annually and is a major cause of noncommunicable disease-related deaths in sub-Saharan Africa (SSA) [2, 3]. In 2010 the prevalence of hypertension among adults was estimated at 1.39 billion persons globally, representing 31% of all adults [4]. In both rural and urban Ghanaian settings, the prevalence of hypertension in adults is reported to range from 19 to 55% [5–7]. This is similar to the estimates in other LMIC, suggesting that Ghana is not spared in the global epidemic [8].

The prevalence of hypertension in the two Kassena-Nankana districts (KNDs) of rural northern Ghana is high. A study from the late 1990s reported a prevalence of 19.3% in the KNDs [9]. Findings from the recent H3Africa AWI-Gen project show an increase in the prevalence of hypertension to 24.8% in adults aged 40–60 years [10]. These findings suggest that the prevalence of hypertension in this rural setting is gradually rising and that hypertension in Ghana is not only an urban problem but a major burden in rural communities as well. In addition to the rising prevalence of hypertension, underdiagnosis and poor control of blood pressure in diagnosed hypertensive subjects have been reported [7, 10, 11]. Fully understanding the determinants of the indices of blood pressure will contribute to holistic management of hypertension.

Although hypertension is diagnosed using systolic blood pressure (SBP) and diastolic blood pressure (DBP), CVD risk may equally be predicted by analyzing pulse pressure (PP) (the difference between SBP and DBP) [12–14], and mean arterial pressure (MAP) (the sum of DBP and one-third of pulse pressure (DBP + (PP/3)) [14]. Pulse pressure is an important predictor in cardiovascular events and predicts the risk of congestive heart failure in the elderly [15], while MAP is the perfusion pressure that is encountered by end organs including liver and kidney [16]. Therefore, combining these blood pressure indices is superior to a single component in predicting CVD, as these may provide additional and holistic pathophysiological information on hypertension, particularly in adults [17]. In combating hypertension, it is important to determine which modifiable risk factors influence these indices so as to inform nonpharmacological measures for targeting the lowering of blood pressure and prevention of CVD in adult men and women.

Studies globally and in urban Ghanaian settings have attributed rising blood pressure to sociodemographic determinants including age and educational status, environmental factors such as exposure to pesticides and alcohol consumption, unfavourable lipid levels, and particular anthropometric indices [18–23]. The question remains as to whether these factors also influence levels of blood pressure among rural Ghanaian adults. An earlier study that looked at the epidemiology of blood pressure in rural northern Ghana investigated mainly anthropometric variables but did not evaluate the influence of other sociodemographic factors or lipid levels on blood pressure indices [9]. Also, the H3Africa AWI-Gen project, in which this study is nested, reported the prevalence of hypertension but did not investigate the factors associated with blood pressure levels in these communities [10]. Furthermore, studies in other African settings have investigated mainly SBP and DBP [24–26] and the factors associated with them, but there is a paucity of data on the factors associated with MAP and PP [27, 28]. This study is the first to report on a wide array of factors associated with SBP, DPB and their derivatives (PP and MAP) in a black African population and therefore provides new knowledge on the association of blood pressure indices with these markers among black Africans and rural northern Ghanaian adults in particular.

## 2. Methods

### 2.1. Study Setting and Population

**Figure 1:**
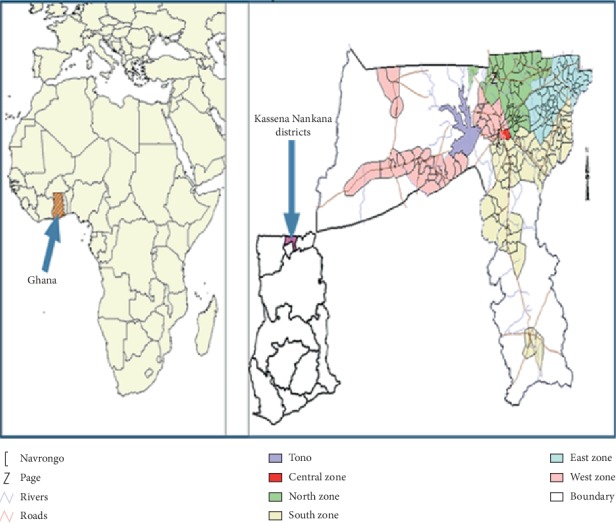
Location of the Kassena-Nankana districts with the zones represented under the Navrongo demographic surveillance system in the upper east region of Ghana.

The study was conducted in the two Kassena-Nankana districts of northern Ghana which are covered by the Navrongo Health and Demographic Surveillance System (NHDSS). The districts are divided into five zones: central, east, west, north, and south zones. These zones cover a total land area of 1675 km^2^ and share a border with Burkina Faso in the northwest. Each of the zones is further divided into clusters, which consist of households (Figure 1). The NHDSS currently monitors 165,000 individuals in 32,000 households. The population density of the districts is 91.5 km^2^ and the major ethnic groups are the Nankana and Kassena. The male to female ratio of the population is 0.92, with an estimated population growth of 0.81% [29].

This study is a part of the H3Africa AWI-Gen project that recruited men and of 40–60 years-of-age [30] mainly in the west and north zones, which is home to the Kassem speaking communities and the east zone which is home to the Nankam speaking communities. Participants of the AWI-Gen project were residents within the study area for at least ten years. Pregnant women and participants who were unable to stand for their height to be measured were excluded from the project.

### 2.2. Study Design and Sampling

The H3Africa AWI-Gen study was a population-based cross-sectional study with stratified random sampling. First, the east, west, north, and south zones were selected from the five zones of the district. Twenty-five clusters each were further randomly sampled from each selected zone using the NHDSS [29]. The list of all men and women aged 40–60 years were generated from each zone. From this list, a sample size of 2200 including 10% for nonresponse was randomly generated with a fraction of this number randomly sampled from each cluster in proportion to the population size of that cluster.

### 2.3. Data Collection

Participants were asked to fast the night prior to blood sampling. On the day of data collection information on sociodemographic factors including age, soscioeconomic status (SES), marital status and educational status, and lifestyle factors such as alcohol consumption, tobacco smoking, pesticide exposure, diet, and physical activity (measured as moderated-to-vigorous physical activity (MVPA)) were collected by trained field workers using a structured questionnaire in a language that the participants understood, using methods that have been described elsewhere [31]. Fasting blood was taken by phlebotomists for lipid analyses. High-density lipoprotein cholesterol (HDL-C), low-density lipoprotein cholesterol (LDL-C), total cholesterol (TC), and total cholesterol were all measured directly [31, 32]. Three measurements of systolic and diastolic blood pressure were taken using a digital sphygmomanometer (Omron M6, Omron, Kyoto, Japan) and the first readings were discarded while an average of the second and third was taken as the measured blood pressure [32]. Anthropometric measures (weight, height, waist and hip circumference, and visceral and subcutaneous adipose tissue thickness) were obtained as described previously [31, 32]. The data were checked for inconsistencies, missingness, and outliers before it was uploaded into the Research electronic data capture (REDCap) platform [33]. After this, further quality control processes were performed on 10% of the data.

### 2.4. Definition of Pesticide Exposure, SBP, and DBP Derivatives

Pesticide exposure was defined by self-reported current working with pesticide or living close to a farm where the pesticide was being used [31]. Pulse pressure (PP) was defined as SBP minus DBP [28], while MAP was defined as the sum of DBP and a third of the PP [17].

### 2.5. Ethics Approval and Consent to Participate

The study was approved by the Human Research Ethics Committee (HREC) of the University of the Witwatersrand (ID No: M12109), the Ghana Health Service Ethics Review Committee (ID No: GHS-ERC:05/05/2015), and the Navrongo Health Research Centre Institutional Review Board (ID No: NHRCIRB178). Community engagement was carried out in the communities where participants were sampled. Individual informed consent, evidenced by a thump-printed or signed informed consent form witnessed by a researcher, was sought from participants before being recruited into the study.

### 2.6. Statistical Analyses

Analyses were performed using Stata 14.2 (StataCorp, College Station, Texas, 77845, US). Due to nonnormal distribution, blood pressure indices (SBP, DBP, MAP, and PP) were presented as medians and interquartile ranges and compared between men and women using Mann–Whitney *U* tests and were log-transformed to normality before regression analyses. Gender-stratified multivariable linear regression analyses were performed to determine the association between log-transformed blood pressure indices and each of the study variables. These variables were included in univariate regression analyses against each of the blood pressure indices, and all those that correlated at *p* < 0.20 were included in multivariable linear regression models for the appropriate blood pressure variable. Multicollinearity was assessed using the variance inflation factor (VIF) and only variables with a VIF <5.0 were included in the models. Association tests at the multivariable regression level were considered statistically significant at 5% significance level.

## 3. Results

A sample size of 2016 was recruited for this study but study participants who did not have data for one or more of the investigated variables were excluded. Therefore the final sample size was 1839 comprising 993 women and 846 men.

### 3.1. Basic Characteristics of the Study Population

The general characteristics of the study population are shown in Table 1. The median age of the population was 51 years with that of women being significantly higher (*p* < 0.001). Formal education (*p* < 0.001), employment (*p*=0.035), marital status (*p* < 0.001), socioeconomic status (*p* < 0.001), current alcohol intake (*p* < 0.001), and current tobacco use (*p* < 0.001) were significantly lower among women than men. However, there was no difference in current smokeless tobacco use between men and women (*p*=0.997). Vegetable servings per day (*p*=0.002) and the number of vendor meals per week (*p* < 0.001) were higher among men than women but there was no difference in the number of fruit servings per day between men and women (*p*=0.094). Women were less physically active and slept longer than men (*p* < 0.001 for both). Except for visceral fat, which was higher in men (*p* < 0.001), all the anthropometric indices (BMI, waist circumference, hip circumference, and subcutaneous fat) were higher among women (*p* < 0.001 for all). Except for HDL-C, which was significantly lower (*p*=0.002) and TC, which was significantly higher (*p*=0.038) in women, there were no significant differences in LDL-C (*p*=0.427) and TG (*p*=0.770) between genders.

The levels of blood pressure indices stratified by gender are shown in Table 2. The median SBP of the study population was 121 (109–135) mmHg with that of men being significantly higher than that of women (*p*=0.007). Similarly, the PP of men was significantly higher than that of women (*p* < 0.001) with a population median PP being 45.0 (38.0–53.0) mmHg. There were, however, no differences in DBP (*p*=0.719) and MAP (*p*=0.340) between men and women.

### 3.2. Factors Associated with Blood Pressure Indices

The factors associated with blood pressure indices (SBP, DBP, PP, and MAP) were stratified by gender and the results are shown in Tables 3–6. The adjusted *β*-coefficients shown in each table are unstandardised and are those derived from multivariable linear regression models that included all the variables that gave *p* < 0.20 in the univariate models (Table S1–S3).

Factors associated with SBP are shown in Table 3. Age (*β* = 0.005, *p* < 0.001), male gender (*β* = 0.044, *p* < 0.001), being currently an unmarried woman (*β* = 0.024, *p* < 0.005), subcutaneous fat thickness (*β* = 0.039, *p* < 0.001), and HDL-C (*β* = 0.054, *p* < 0.001) were associated with increased SBP among the total population. Pesticide exposure among women was associated with decreased SBP (*β* = −0.020, *p* < 0.005). Together all these factors contributed to 9.5% of the variance in SBP for the total population.

The factors that were associated with increased DBP among the total population were age (*β* = 0.004; *p* < 0.001), hip circumference (*β* = 0.020; *p* < 0.05), visceral fat thickness (*β* = 0.010; *p* < 0.05), subcutaneous fat thickness (*β* = 0.076; *p* < 0.001), and HDL-C (*β* = 0.064; *p* < 0.001). Among women, being currently unmarried (*β* = 0.023; *p* < 0.05) was associated with increased DBP, while pesticide exposure (*β* = −0.028; *p* < 0.005) was associated with decreased DBP. All these factors contributed to a 10.2% variance in DBP in the total population (Table 4).

Factors associated with MAP in the study population are shown in Table 5. Age (*β* = 0.004; *p* < 0.001), visceral fat thickness (*β* = 0.012; *p* < 0.001), subcutaneous fat thickness (*β* = 0.035; *p* < 0.001), and HDL-C level (*β* = 0.057; *p* < 0.001) were associated with increased MAP in the total population while among women pesticide exposure (*β* = −0.031; *p* < 0.005) was associated with decreased MAP and being unmarried was associated with a higher MAP (*β* = 0.030; *p* < 0.005). In men, hip circumference was associated with a higher MAP (*β* = 0.019; *p* < 0.05). These factors accounted for 9.5% variance in MAP in the total population.

Age (*β* = 0.009; *p* < 0.001), male gender (*β* = 0.079; *p* < 0.001), Nankana ethnicity (*β* = 0.042; *p* < 0.005), being an unmarried woman (*β* = 0.044; *p* < 0.005), and vendor meal consumption per month among women (*β* = 0.0004; *p* < 0.050) were associated with increased PP. Pesticide exposure among women (*β* = −0.042; *p* < 0.050) and fruit servings per week among men (*β* = −0.001; *p* < 0.050) were associated with decreased PP. These factors explain 5.3% of the variance in PP in the total population.

## 4. Discussion

The key findings of this study were that all the blood pressure indices increased with increasing age among the total study population. Male gender was associated with higher SBP and PP. Nankana ethnicity was associated with higher PP in men. Women who were currently unmarried had higher levels of all the blood indices compared to married women. Pesticide exposure was associated with reduced levels of all the measured blood pressure indices in women, but not in men. Vendor meal intake in women was associated with higher levels of PP while fruit consumption in men was associated with lower PP levels. Increased abdominal subcutaneous fat was associated with higher DBP and MAP levels in both men and women and with higher SBP levels in men. Increased visceral fat thickness was associated with higher DBP and MAP levels in the total population. In men, increased hip circumference was associated with higher DBP and MAP levels. Increased HDL-C levels were associated with higher SBP, DBP, and MAP in both men and women and higher PP in men.

The increase in blood pressure indices with increasing age in the study population confirms earlier findings in the study area [9], other rural settings in Ghana [34], and elsewhere in sub-Saharan Africa [25, 35, 36]. Increasing levels of blood pressure indices with ageing is attributed to several pathophysiological factors such as decreased baroreceptor sensitivity, increased responsiveness to sympathetic nervous system stimuli, and altered sodium metabolism resulting in increased salt sensitivity [37, 38]. Ageing is associated with changes in large artery stiffness and increased peripheral vascular resistance resulting in increasing SBP and DBP, respectively [39, 40].

The higher SBP in men compared to women observed in our study is consistent with earlier results in the study setting [9] and in other African settings [27, 28, 41]. While the higher PP among men in our study resonates with some of these African findings [28] other results showed no gender difference in PP [27]. The higher PP in the Nankana ethnic group suggests an influence of ethnicity on blood pressure levels and this is consistent with findings elsewhere [42, 43]. The ethnic differences in blood pressure may be attributed to the potential influence of genetic factors [42].

Our study has shown that being an unmarried woman was associated with high levels of blood pressure indices. This is similar to findings in African American [44] and Arab communities [45] where single or widowed women had higher blood pressure compared to married women. Being married is reported to be protective against adverse health outcomes [46] including CVDs [46, 47]. The higher levels of blood pressure indices among unmarried women may be attributed partly to stress associated with a lack of social support [48].

The reason for the lower levels of all the blood pressure indices with pesticide exposure among women is not known and further studies, involving the type and blood concentrations of pesticides, are required to explain the possible mechanisms underlying this observation. There are mixed findings on blood pressure and pesticide exposure in the literature. Thus, some studies have reported higher blood pressure in response to pesticide exposure [49, 50] while others have reported contrary findings [51, 52].

The negative association of PP with fruit consumption among men and the positive association of vendor meal consumption with PP among men in the study population support previous findings [53–55] and confirm that healthier diet choices can reduce the burden of hypertension [56] and reduce CVD risk [57].

In this population subcutaneous fat thickness was associated with higher SBP levels in men while both subcutaneous and visceral fat thickness, in both genders, were associated with higher DBP and MAP levels with subcutaneous fat being the adiposity marker with the strongest association. There are many proposed mechanisms for the association of obesity with hypertension [58] with one of the major hypotheses involving the effect of leptin on sympathetic neural activation [59]. This is relevant to the findings in the current study as subcutaneous adipose tissue has been shown to be the main source of leptin in females [60] and this fat depot is the primary determinant of serum leptin levels [61]. However, leptin was not measured in the present study and therefore we are not able to test the hypothesis that leptin mediates the effect of subcutaneous adipose tissue on blood pressure parameters. The positive association if hip circumference with DBP was unexpected as the hip is known to be protective against CVD risk [62, 63]. Further studies in this population are therefore required to confirm this.

The increase in blood pressure indices with increasing HDL-C levels in this population is supported by data from Burkina Faso [64]. This raises the question of whether low HDL-C is truly a reliable predictor of CVD risk in rural northern Ghana.

The strength of this study is that it is the first to investigate the effect of a wide range of factors on an array of blood pressure indices in a rural African adult population. It therefore provides new knowledge on the factors associated with high blood pressure in black Africans. However the study is not without limitations. Causality cannot be inferred due to the cross-sectional design of the study. Interpretation of the results from the questionnaire data should be taken with caution due to possible recall bias from the self-reported responses of the participants. Also, despite the wide range of factors investigated in our study including vendor meal, fruit, and vegetable consumption data on salt which is a known risk factor of hypertension [65] was not investigated. Another limitation of the study was that data on the composition and extent or duration of exposure of the pesticides were not collected.

## 5. Conclusion

Our study demonstrates that SBP, DBP, MAP, and PP are driven by a number of modifiable risk factors which have important implications for possible public health intervention projects. The positive association of subcutaneous fat with blood pressure indices may be indicative of an emerging problem in this rural population and calls for interventions to curb obesity among the study population. Lifestyle interventions specifically fruit intake which is associated with reduced PP among men should be adopted, and vendor meal consumption which is associated with higher PP among women should be avoided to reduce CVD risk among the study population. The higher BP indices among unmarried women call for the need for further studies to identify appropriate healthcare interventions targeted at widows, single women, and women with less social support in rural communities. The lower blood pressure indices associated with pesticide exposure among women calls for further evaluation of the use of pesticides in the community, since these may pose the risk of hypotension among women. The ethnic difference in blood pressure indices in the study population may suggest the potential influence of genetic factors.

## Figures and Tables

**Table 1:** General characteristics of the rural adult Ghanaian study population stratified by gender and for the combined population.

Variables	Men (*n* = 846, 46%)	Women (*n* = 993, 54%)	Total (*N* = 1839)	*p* value
Age (years)	50 (40–66)	52 (40–60)	51 (40–60)	<0.001

Ethnicity				
Kassena	439 (51.9)	516 (52.0)	955 (51.9)	<0.001
Nankana	392 (46.3)	401 (40.4)	793 (43.1)	
Minority ethnic groups	15 (1.77)	76 (7.65)	91 (4.95)	

Educational status				
No formal education	517 (61.1)	768 (77.3)	1285 (69.9)	<0.001
Formal education	329 (38.9)	225 (22.7)	554 (30.1)	

Employment status				
Unemployed	292 (34.5)	390 (39.3)	682 (37.1)	0.035
Employed	554 (65.5)	603 (60.7)	1157 (62.9)	

Marital status				
Currently married	717 (84.8)	632 (63.7)	1349 (73.4)	<0.001
Currently unmarried	129 (15.3)	361 (36.4)	490 (26.6)	

Household SES				
Low SES	625 (73.8)	817 (82.3)	430 (23.4)	<0.001
High SES	221 (26.1)	176 (17.7)	397 (21.6)	

Fruit intake (servings/week)	7 (0–7)	7 (0–7)	7 (0–7)	0.094
Vegetable intake (servings/week)	21.0 (14.0–35.0)	21.0 (14.0–28.0)	21.0 (14.0–28.0)	0.002
Vendor meals (times/month)	0.003 (0.00–60.0)	0.00 (0.00–30.0)	0.00 (0.00–60.0)	<0.001

Alcohol intake				
No alcohol intake	291 (34.4)	606 (60.6)	893 (48.6)	
Current alcohol intake	555 (65.6)	391 (39.4)	946 (51.4)	<0.001

Tobacco smoking				
No smoking	661 (78.1)	979 (98.6)	1640 (89.2)	
Current smoking	185 (21.9)	14 (1.4)	199 (10.9)	<0.001

Smokeless tobacco				
No smokeless tobacco use	760 (89.8)	892 (89.8)	1652 (89.8)	
Current smokeless tobacco use	86 (10.2)	101 (10.2)	187 (10.2)	0.997

Pesticide exposure	509 (60.2)	497 (50.1)	1006 (54.7)	<0.001
Physical activity				
Less physically active	80 (9.46)	182 (18.33)	262 (14.25)	<0.001
More physically active	766 (90.54)	811 (81.67)		

MVPA (hours/week)	41.0 (17.0–5.00)	28.0 (5.00–44.0)	34.5 (9.00–48.0)	<0.001
Sleeping (hours/week)	56 (49–61)	56 (54–63)	56 (49–63)	<0.001
BMI (kg/m^2^)	20.5 (19.0–22.3)	21.5 (19.7–24.0)	20.5 (19.0–22.3)	<0.001
Hip (cm)	8.30 (7.90–8.80)	8.80 (8.30–9.40)	8.30 (7.90–8.80)	<0.001
Waist (cm)	7.20 (6.80–7.70)	7.50 (7.10–8.20)	7.40 (6.90–7.90)	<0.001
HDL-C (mmol/l)	1.14 (0.90–1.40)	1.08 (0.89–1.31)	1.10 (0.90–1.35)	0.002
LDL-C (mmol/l)	1.58 (1.12–2.16)	1.61 (1.19–2.13)	1.60 (1.16–2.14)	0.427
TC (mmol/l)	3.13 (2.59–3.70)	3.20 (2.69–3.82)	3.16 (2.63–3.78)	0.038
TG (mmol/l)	0.56 (0.43–0.73)	0.55 (0.43–0.73)	0.55 (0.43–0.73)	0.770
Visceral fat (cm)	4.03 (3.29–4.83)	3.32 (2.75–4.10)	3.63 (2.99–4.50)	<0.001
Subcutaneous fat (cm)	0.70 (0.56–0.87)	1.01 (0.74–1.45)	0.83 (0.62–1.18)	<0.001

Data shown as *n* (%) or median (interquartile range); MVPA: moderate-to-vigorous physical activity.

**Table 2:** Median levels of blood pressure indices in the rural adult Ghanaian study population stratified by gender and in the combined population.

Variable	Men	Women	Total	*p* value
SBP (mmHg)	122 (111–136)	120 (108–135)	121 (109–135)	0.007
DBP (mmHg)	75.5 (68.0–84.0)	75.5 (68.5–84.5)	75.5 (68.0–84.5)	0.719
PP (mmHg)	46.5 (40.5–54.0)	43.5 (37.0–52.5)	45.0 (38.0–53.0)	<0.001
MAP (mmHg)	90.6 (82.7–101)	90.0 (81.7–101)	90.3 (75.7–101)	0.340

Data given as median (interquartile range).

**Table 3:** Multivariable linear regression analysis of factors associated with SBP among men and women and the total population.

Variable	Women	Men	Total
	*β*-coefficient (95% CI)	*β*-coefficient (95% CI)	*β*-coefficient (95% CI)
Age (years)	0.006 (0.004, 0.004)^*∗∗∗*^	0.005 (0.003, 0.007)^*∗∗∗*^	0.005 (0.004, 0.007)^*∗∗∗*^
Male	—	—	0.044 (0.026, 0.062)^*∗∗∗*^
High SES^1^	—	−0.007 (−0.033, 0.018)	−0.005 (−0.024, 0.014)
Currently unmarried	0.038 (0.016, 0.060)^*∗∗*^	—	0.024 (0.007, 0.041)^*∗∗*^
Past or current smoker^2^	—	−0.019 (−0.041, 0.003)	—
Used smokeless tobacco	0.016 (−0.019, 0.051)	—	0.015 (−0.009, 0.040)
Pesticide exposure	−0.034 (−0.055, −0.013)^*∗∗*^	—	−0.020 (−0.036, −0.005)^*∗∗*^
MVPA (hours/week)	−0.013 (−0.027, 0.002)	—	−0.010 (−0.021, 0.002)
Fruit (servings/week)	−0.0002 (−0.001, 0.001)	—	−0.0004 (−0.001, 0.0003)
BMI (kg/m^2^)	0.005 (−0.001, 0.010)	3.96*e*−06 (−0.004, 0.004)	0.002 (−0.001, 0.006)
Waist (cm)	0.015 (−0.007, 0.036)	−0.002 (−0.020, 0.016)	0.006 (−0.007, 0.020)
Hip (cm)	−0.005 (−0.022, 0.012)	0.018 (−0.004, 0.036)	0.006 (−0.007, 0.018)
Visceral fat (cm)	0.003 (−0.008, 0.014)	0.008 (−0.001, 0.017)	0.006 (−0.001, 0.013)
Subcutaneous fat (cm)	0.026 (−0.003, 0.055)	0.053 (0.021, 0.086)^*∗∗*^	0.039 (0.018, 0.060)^*∗∗∗*^
HDL-C (mmol/l)	0.055 (0.021, 0.089)^*∗∗*^	0.063 (0.034, 0.091)^*∗∗∗*^	0.054 (0.032, 0.076)^*∗∗∗*^
LDL-C (mmol/l)	—	0.001 (−0.015, 0.017)	−0.011 (−0.023, 0.001)
TC (mmol/l)	−0.003 (−0.015, 0.010)	0.003 (−0.013, 0.018)	0.007 (−0.004, 0.018)
TG (mmol/l)	−0.032 (−0.064, 0.001)	0.013 (−0.006, 0.031)	0.003 (−0.014, 0.020)

All blood pressure components were log-transformed; ^1^SES was coded as those with highest vs. those with lowest SES; ^2^Smoking status was coded as those who are current or past smokers vs. those who never smoked; ^*∗∗∗*^*p* value < 0.001; ^*∗∗*^*p* value < 0.005; ^*∗*^*p* value < 0.05.

**Table 4:** Multivariable linear regression analysis of factors associated with DBP among men and women and the total population.

Variable	Women	Men	Total
	*β*-coefficient (95% CI)	*β*-coefficient (95% CI)	*β*-coefficient (95% CI)
Age (years)	0.003 (0.001, 0.005)^*∗∗*^	0.004 (0.002, 0.006)^*∗∗∗*^	0.004 (0.002, 0.006)^*∗∗∗*^
Nankana ethnicity	0.001 (−0.020, 0.021)	—	−0.006 (−0.030, 0.019)
Some formal education^1^	0.018 (−0.005, 0.041)	—	0.008 (−0.014, 0.030)
Employed	—	−0.017 (−0.040, 0.005)	—
High SES^2^	−0.010 (−0.037, −0.016)	−0.004 (−0.030, 0.022)	−0.010 (−0.036, 0.017)
Currently unmarried	0.023 (0.003, 0.043)^*∗*^	—	−0.001 (−0.031, 0.029)
Past or current smoker^3^	−0.021 (−0.074, 0.032)	—	−0.009 (−0.032, 0.014)
Pesticide exposure	−0.028 (−0.048, −0.009)^*∗∗*^	—	−0.007 (−0.031, −0.016)
MVPA (hours/week)	−0.008 (−0.022, 0.006)	−0.019 (−0.039, 0.002)	−0.018 (−0.038, 0.003)
BMI (kg/m^2^)	0.004 (−0.001, 0.009)	−0.001 (−0.005, 0.004)	−0.001 (−0.005, 0.004)
Waist (cm)	0.020 (−0.0001, 0.040)	0.002 (−0.016, 0.021)	0.001 (−0.018, 0.020)
Hip (cm)	−0.005 (−0.010, 0.021)	0.020 (0.002, 0.039)^*∗*^	0.020 (0.001, 0.038)^*∗*^
Visceral fat (cm)	0.003 (−0.007, 0.013)	0.009 (−0.001, 0.018)	0.010 (0.0001, 0.019)^*∗*^
Subcutaneous fat (cm)	0.036 (0.009, 0.062)^*∗∗*^	0.078 (0.045, 0.111)^*∗∗∗*^	0.076 (0.043, 0.109)^*∗∗∗*^
HDL-C (mmol/l)	0.055 (0.023, 0.086)^*∗∗*^	0.061 (0.032, 0.091)^*∗∗∗*^	0.064 (0.033, 0.094)^*∗∗∗*^
LDL-C (mmol/l)	−0.017 (−0.033, 0.0002)	−0.003 (−0.020, 0.013)	−0.003 (−0.020, 0.014)
TC (mmol/l)	0.008 (−0.007, 0.022)	0.003 (−0.013, 0.019)	0.001 (−0.016, 0.018)
TG (mmol/l)	−0.016 (−0.048, 0.016)	0.019 (−0.001, 0.038)	0.019 (−0.001, 0.038)

All blood pressure components were log-transformed; ^1^education was coded as some formal education vs. no education; ^2^SES was coded as those with highest vs. those with lowest SES; ^3^smoking status was coded as those who are current or past smokers vs. those who never smoked; ^*∗∗∗*^*p* value < 0.001; ^*∗∗*^*p* value < 0.005; ^*∗*^*p* value < 0.05.

**Table 5:** Multivariable linear regression analysis of factors associated with MAP among men and women and the total population.

Variable	Women	Men	Total
	*β*-coefficient (95% CI)	*β*-coefficient (95% CI)	*β*-coefficient (95% CI)
Age (years)	0.004 (0.002, 0.006)^*∗∗*^	0.004 (0.003, 0.006)^*∗∗∗*^	0.004 (0.003, 0.005)^*∗∗∗*^
Some formal education^1^	0.012 (−0.011, 0.036)	—	—
High SES^2^	−0.014 (−0.041, −0.013)	−0.008 (−0.033, 0.017)	0.002 (−0.016, 0.020)
Currently unmarried	0.030 (0.010, 0.051)^*∗∗*^	—	0.011 (−0.016, 0.020)
Past or current smoker^3^	—	−0.013 (−0.035, 0.008)	—
Pesticide exposure	−0.031 (−0.050, −0.011)^*∗∗*^	—	−0.016 (−0.030, −0.002)^*∗*^
MVPA (hours/week)	−0.011 (−0.024, 0.003)	—	−0.009 (−0.020, 0.002)
BMI (kg/m^2^)	0.005 (−0.001, 0.009)	−0.0003 (−0.005, 0.004)	0.002 (−0.002, 0.005)
Waist (cm)	0.016 (−0.004, 0.036)	0.0001 (−0.018, 0.018)	0.008 (−0.005, 0.022)
Hip (cm)	0.001 (−0.015, 0.016)	0.019 (0.001, 0.036)^*∗*^	0.005 (−0.007, 0.016)
Visceral fat (cm)	0.003 (−0.008, 0.013)	0.009 (−0.00003, 0.018)	0.012 (0.006, 0.018)^*∗∗∗*^
Subcutaneous fat (cm)	0.034 (0.007, 0.060)^*∗*^	0.066 (0.035, 0.098)^*∗∗∗*^	0.035 (0.016, 0.054)^*∗∗∗*^
HDL-C (mmol/l)	0.058 (0.026, 0.089)^*∗∗∗*^	0.062 (0.033, 0.090)^*∗∗∗*^	0.057 (0.036, 0.078)^*∗∗∗*^
LDL-C (mmol/l)	—	−0.001 (−0.017, 0.015)	−0.010 (−0.021, 0.001)
TC (mmol/l)	−0.001 (−0.013, 0.011)	0.002 (−0.013, 0.018)	0.006 (−0.004, 0.016)
TG (mmol/l)	−0.030 (−0.060, 0.00004)	0.017 (−0.001, 0.036)	0.008 (−0.008, 0.024)

All blood pressure components were log-transformed; ^1^education was coded as some formal education vs. no education; ^2^SES was coded as those with highest vs. those with lowest SES; ^3^smoking status was coded as those who are current or past smokers vs. those who never smoked; ^*∗∗∗*^*p* value < 0.001; ^*∗∗*^*p* value < 0.005; ^*∗*^*p* value < 0.05.

**Table 6:** Multivariable linear regression analysis of factors associated with PP among men and women and the total population.

Variable	Women	Men	Total
	*β*-coefficient (95% CI)	*β*-coefficient (95% CI)	*β*-coefficient (95% CI)
Age (years)	0.010 (0.007, 0.013)^*∗∗*^	0.006 (0.003, 0.009)^*∗∗∗*^	0.009 (0.007, 0.011)^*∗∗∗*^
Male gender	—	—	0.079 (0.050, 0.109)^*∗∗∗*^
Nankana ethnicity	—	0.037 (0.004, 0.071)^*∗*^	0.042 (0.018, 0.066)^*∗∗*^
High SES^2^	−0.025 (−0.070, 0.020)	—	—
Currently unmarried	0.054 (0.020, 0.088)^*∗∗*^	—	0.044 (0.018, 0.070)^*∗∗*^
Some formal education^1^	0.001 (−0.038, 0.041)	—	—
Past or current smoker^2^	—	−0.010 (−0.031, 0.011)	−0.010 (−0.030, 0.011)
Used smokeless tobacco	0.023 (−0.031, 0.077)	0.026 (−0.024, 0.077)	0.023 (−0.014, 0.061)
Pesticide exposure	−0.042 (−0.072, −0.009)^*∗*^	—	−0.011 (−0.035, 0.013)
Vendor (meals/month)	0.0004 (0.00002, 0.0008)^*∗*^	—	—
Fruit (servings/week)	—	−0.001 (−0.003, −0.00001)^*∗*^	−0.001 (−0.002, 0.0003)
MVPA (hours/week)	−0.019 (−0.042, 0.004)	0.018 (−0.011, 0.047)	−0.009 (−0.027, 0.009)
Sleeping (hours/week)	—	0.001 (−0.001, 0.003)	—
Hip (cm)	0.005 (−0.013, 0.022)	—	0.011 (−0.002, 0.024)
Visceral fat (cm)	—	0.011 (−0.001, 0.024)	—
HDL-C (mmol/l)	0.044 (−0.003, 0.091)	0.044 (0.001, 0.087)^*∗*^	0.048 (0.014, 0.082)^*∗*^
LDL-C (mmol/l)	—	0.005 (−0.020, 0.029)	—
TC (mmol/l)	—	0.017 (−0.007, 0.042)	0.007 (−0.007, 0.021)

All blood pressure components were log-transformed; ^1^education was coded as some formal education vs. no education; ^2^smoking status was coded as those who are current or past smokers vs. those who never smoked; ^*∗∗∗*^*p* value < 0.001; ^*∗∗*^*p* value < 0.005; ^*∗*^*p* value < 0.05.

## Data Availability

All data analyzed during this study are included within this published article in the supplementary files.

## Supplementary Materials

Supplementary Materials Table S1: univariable linear regression analysis of factors associated with SBP, DBP, MAP, and PP in the total population. Table S2: univariate linear regression analysis of factors associated with SBP, DBP, MAP, and PP among rural northern Ghanaian women. Table S3: univariate linear regression analysis of factors associated with SBP, DBP, MAP, and PP among rural northern Ghanaian men.
