# Supplementary material for: Blood Pressure Indices and Associated Risk Factors in a Rural West African Adult Population: Insights from an AWI-Gen Substudy in Ghana
Source: Int J Hypertens. 2020 Apr 26;2020:4549031. doi: 10.1155/2020/4549031 (PMC7201512; doi:10.1155/2020/4549031)
Supplement: Supplementary Materials — Table S1: univariable linear regression analysis of factors associated with SBP, DBP, MAP, and PP in the total population. Table S2: univariate linear regression analysis of factors associated with SBP, DBP, MAP, and PP among rural northern Ghanaian women. Table S3: univariate linear regression analysis of factors associated with SBP, DBP, MAP, and PP among rural northern Ghanaian men. [file 4549031.f1.docx]

**Table S1: Univariable linear regression analysis of factors associated with SBP, DBP, MAP and PP in the total population**

| Independent variables | SBP | DBP | MAP | PP |
| --- | --- | --- | --- | --- |
|  | Unstandardized β-Coefficient (95%CI) | | | |
| Age (years) | 0.005 (0.004, 0.006)**** | 0.003 (0.001, 0.004)**** | 0.004 (0.003, 0.005)**** | 0.009 (0.007, 0.011)**** |
| Male gender | 0.016 (0.001, 0.032)** | -0.003 (-0.018, 0.012) | 0.006 (-0.009, 0.020) | 0.051 (0.028, 0.074)**** |
| Nankana ethnicity | 0.009 (-0.006, 0.025) | -0.016 (-0.031, -0.001)** | -0.005 (-0.019, 0.010) | 0.020 (0.001, 0.040)** |
| Some formal education^1^ | 0.008 (-0.009, 0.025) | 0.018 (0.002, 0.034)** | -0.014 (-0.002, 0.029) | -0.010 (-0.035, 0.015) |
| Employed | -0.0003 (-0.016, 0.015) | -0.005 (-0.020, 0.010) | -0.003 (-0.018, 0.012) | 0.008 (-0.016, 0.032) |
| Currently unmarried | 0.024 (0.007, 0.041)*** | 0.013(-0.003, 0.030)* | 0.018 (0.002, 0.034)** | 0.037 (0.012, 0.063)*** |
| High SES^2^ | 0.014 (-0.005, 0.032)* | 0.027 (0.009, 0.044)*** | 0.021 (0.003, 0.038)** | -0.009 (-0.037, 0.018) |
| Past or current smoker^3^ | -0.0004 (-0.017, 0.016) | -0.012 (-0.027, 0.004)* | -0.007 (-0.022, 0.009) | 0.021 (-0.004, 0.045)* |
| Used smokeless tobacco | 0.023 (-0.002, 0.048)* | 0.005 (-0.019, 0.029) | 0.013 (-0.011, 0.037) | 0.052 (0.014, 0.090)*** |
| Past or current drinker^4^ | -0.008 (-0.013, 0.029) | 0.008 (-0.011, 0.029) | 0.008 (-0.012, 0.028) | 0.008 (-0.025, 0.040) |
| Pesticide exposure | -0.024 (-0.039, -0.008)*** | -0.015 (-0.030, -0.001)** | -0.019 (-0.033, -0.005)*** | -0.033 (-0.056, -0.010)*** |
| Fruit (servings/day) | -0.004 (-0.009, 0.0003)* | -0.002 (-0.006, 0.003) | -0.0004 (-0.001, -0.0002)* | -0.001 (-0.002, -0.0002)** |
| Vegetable (servings/day) | 0.001 (-0.004, 0.006) | -0.0002 (-0.005, 0.005) | 0.0001 (-0.001, 0.001) | 0.001 (-0.001, 0.002) |
| Physically active | 0.004 (-0.001, 0.010)* | 0.003 (-0.002, 0.008) | 0.0001 (-0.0003, 0.0003) | 0.0002 (-0.00002, 0.0005)* |
| MVPA (hours/week) | -0.0002 (-0.0005, 0.0001)* | -0.0002 (-0.004, 0.00002)* | -0.015 (-0.027, -0.004)*** | -0.007 (-0.025, 0.011) |
| Sleeping (hours/night) | 0.001 (-0.005, 0.006) | -0.0003 (-0.006, 0.005) | 0.00003 (-0.001, 0.001) | 0.0003 (-0.001, 0.002) |
| BMI (kg/m^2^) | 0.005 (0.003, 0.007)**** | 0.009 (0.007, 0.011)**** | 0.007 (0.005, 0.009)**** | -0.001 (-0.005, 0.002) |
| Waist (cm) | 0.023 (0.015, 0.032)**** | 0.037 (0.030, 0.045)**** | 0.031 (0.023, 0.039)**** | -0.002 (-0.014, 0.011) |
| Hip (cm) | 0.015 (0.007, 0.023)**** | 0.031 (0.023, 0.039)**** | 0.024 (0.016, 0.031)**** | -0.012 (-0.024, 0.0001)* |
| Visceral fat (cm) | 0.017 (0.010, 0.023)**** | 0.019 (0.013, 0.026)**** | 0.018 (0.012, 0.024)**** | 0.011 (0.002, 0.021)** |
| Subcutaneous fat (cm) | 0.047 (0.032, 0.062)**** | 0.076 (0.062, 0.090)**** | 0.063 (0.049, 0.077)**** | -0.005 (-0.028, 0.017) |
| HDL-C (mmol/l) | 0.058 (0.038, 0.077)**** | 0.054 (0.034, 0.073)**** | 0.055 (0.036, 0.074)**** | 0.066 (0.035, 0.096)**** |
| LDL-C (mmol/l) | 0.011 (0.002, 0.021)** | 0.015 (0.005, 0.024)**** | 0.013 (0.004, 0.022)*** | 0.006 (-0.008, 0.021) |
| TC (mmol/l) | 0.019 (0.011, 0.028)**** | 0.022 (0.014, 0.029)**** | 0.021 (0.013, 0.028)**** | 0.015 (0.003, 0.028)** |
| TG (mmol/l) | 0.017 (0.0005, 0.034)** | 0.027 (0.011, 0.043)**** | 0.023 (0.007, 0.039)*** | 0.0003 (-0.025, 0.026) |

All blood pressure indices were log-transformed; 1education was coded as some formal education vs. no education; 2SES was coded as those with highest vs. those with lowest SES; 3smoking status was coded as those who are current or past smokers vs. those who never smoked; 4alcohol intake was coded as those who had ever drunk alcohol vs. those who had never drunk; *p value<0.2; **p value<0.05; ***p value<0.01; ****p value<0.001

**Table S2: Univariate linear regression analysis of factors associated with SBP, DBP, MAP and PP among rural northern Ghanaian women**

| Independent variables | SBP | DBP | MAP | PP |
| --- | --- | --- | --- | --- |
|  | Unstandardized β-Coefficient (95%CI) | | | |
| Age (years) | 0.006 (0.004, 0.007)**** | 0.002 (0.0004, 0.004)** | 0.004 (0.002, 0.005)**** | 0.011 (0.009, 0.014)**** |
| Nankana ethnicity | 0.006 (-0.016, 0.028) | -0.018(-0.038, 0.002)* | -0.007 (-0.027, 0.013) | 0.013 (-0.013, 0.040) |
| Some formal education^1^ | 0.006 (-0.020, 0.032) | 0.027 (0.003, 0.050)** | 0.018 (-0.006, 0.041)* | -0.030 (-0.070, 0.010)* |
| Employed | 0.004 -0.018, 0.026) | 0.004 (-0.016, 0.024) | -0.004 (-0.016, 0.024) | 0.006 (-0.028, 0.040) |
| Currently unmarried | 0.049 (0.027, 0.071)**** | 0.027(-0.006, 0.047)** | 0.037 (0.016, 0.057)**** | 0.082 (0.048, 0.116)*** |
| High SES^2^ | 0.006 (-0.022, 0.034) | 0.031 (0.005, 0.057)** | 0.021 (-0.003, 0.045)* | -0.039 (-0.083, 0.004)* |
| Past or current smoker^3^ | -0.038 (-0.098, 0.022) | -0.045 (-0.100, 0.010)* | -0.042 (-0.097, 0.013) | -0.021 (-0.114, 0.072) |
| Used smokeless tobacco | 0.025 (-0.011, 0.060)* | 0.002 (-0.031, 0.035) | 0.012 (-0.020, 0.045) | 0.063 (0.008, 0.117)** |
| Past or current drinker^4^ | -0.004 (-0.030, 0.023) | 0.006 (-0.018, 0.030) | 0.002 (-0.022, 0.026) | -0.017 (-0.058, 0.023) |
| Pesticide exposure | -0.039 (-0.060, -0.018)**** | -0.024 (-0.044, -0.004)** | -0.031 (-0.051, -0.011)*** | -0.059 (-0.092, -0.026)**** |
| Fruit (servings/week) | -0.001 (-0.002, 0.0003)* | -0.0005 (-0.001, 0.0004) | -0.001 (-0.001, 0.0003) | -0.001 (-0.002, 0.001) |
| Vegetable servings/week | 0.0004 (-0.001, 0.001) | 0.0003 (-0.0006, 0.001) | 0.0004 (-0.001, 0.001) | 0.001 (-0.001, 0.002) |
| Vendor (meals/month) | 0.0002 (-0.0001, 0.0004) | 0.0001 (-0.0002, 0.0003) | 0.0001 (-0.0001, 0.0004) | 0.0003 (-0.0001, 0.001)* |
| Physically active | -0.021 (-0.037, -0.006)*** | -0.0002 (-0.004, 0.00002)* | -0.018 (-0.033, -0.004)** | -0.028 (-0.051, -0.004)** |
| Sleeping (hours/week) | - 4.16e-07 (-0.001, 0.0.001) | -0.0003 (-0.001, 0.0008) | -0.0001 (-0.001, 0.001) | 0.001 (-0.001, 0.002) |
| BMI (kg/m^2^) | 0.006 (0.004, 0.009)**** | 0.010 (0.008, 0.013)**** | 0.009 (0.006, 0.011)**** | -0.001 (-0.005, 0.003) |
| Waist (cm) | 0.029 (0.018, 0.040)**** | 0.044 (0.034, 0.054)**** | 0.037 (0.027, 0.047)**** | 0.001 (-0.016, 0.019) |
| Hip (cm) | 0.016 (0.006, 0.027)*** | 0.034 (0.025, 0.044)**** | 0.026 (0.014, 0.040)**** | -0.014 (-0.031, 0.002)* |
| Visceral fat (cm) | 0.017 (0.008, 0.027)**** | 0.019 (0.013, 0.026)**** | 0.021 (0.012, 0.030)**** | 0.005 (-0.010, 0.020) |
| Subcutaneous fat (cm) | 0.055 (0.035, 0.075)**** | 0.081 (0.063, 0.098)**** | 0.070 (0.052, 0.087)**** | 0.006 (-0.025, 0.037) |
| HDL-C (mmol/l) | 0.051 (0.019, 0.082)*** | 0.053 (0.024, 0.082)**** | 0.052 (0.023, 0.081)**** | 0.048 (-0.001, 0.096)* |
| LDL-C (mmol/l) | 0.004 (-0.010, 0.018) | 0.011 (-0.002, 0.024)**** | 0.008 (-0.005, 0.021) | -0.009 (-0.031, 0.013) |
| TC (mmol/l) | 0.018 (0.007, 0.030)*** | 0.022 (0.011, 0.033)**** | 0.020 (0.010, 0.031)**** | 0.011 (-0.007, 0.029) |
| TG (mmol/l) | 0.026 (-0.006, 0.057)* | 0.037 (0.008, 0.066)** | 0.032 (0.003, 0.061)** | 0.0007 (-0.041, 0.056) |

All blood pressure indices were log-transformed; 1education was coded as some formal education vs. no education; 2SES was coded as those with highest vs. those with lowest SES; 3smoking status was coded as those who are current or past smokers vs. those who never smoked; 4alcohol intake was coded as those who had ever drunk alcohol vs. those who had never drunk; *p value<0.2; **p value<0.05; ***p value<0.01; ****p value<0.001

**Table S3: Univariate linear regression analysis of factors associated with of SBP, DBP, MAP and PP among rural northern Ghanaian men**

| Independent variables | SBP | DBP | MAP | PP |
| --- | --- | --- | --- | --- |
|  | Unstandardized β-Coefficient (95%CI) | | | |
| Age (years) | 0.005 (0.003, 0.007)**** | 0.003 (0.001, 0.005)*** | 0.004 (0.002, 0.006)**** | 0.007 (0.004, 0.009)**** |
| Nankana ethnicity | 0.011 (-0.024, 0.017) | -0.013 (-0.035, 0.009) | -0.002 (-0.023, 0.019) | 0.038 (0.009, 0.066)** |
| Some formal education^1^ | 0.004 (-0.018, 0.026) | 0.013 (-0.009, 0.036) | 0.009 (-0.012, 0.031) | -0.011 (-0.043, 0.020) |
| Employed | -0.008 (-0.031, 0.014) | -0.015 (-0.038, 0.008)* | -0.012 (-0.034, 0.010) | 0.004 (-0.028, 0.037) |
| Currently unmarried | -0.010 (-0.040, 0.020) | -0.015 (-0.045, 0.016) | -0.013 (-0.042, 0.017) | -0.005 (-0.048, 0.038) |
| High SES^2^ | 0.017 (-0.007, 0.041)* | 0.024 (-0.001, 0.049)* | 0.021 (-0.003, 0.045)* | 0.005 (-0.030, 0.040) |
| Past or current smoker^3^ | -0.018 (-0.041, 0.004)* | -0.012 (-0.035, 0.011) | -0.015 (-0.037, 0.007)* | -0.028 (-0.060, 0.005)* |
| Used smokeless tobacco | 0.021 (-0.015, 0.056) | 0.009 (-0.027, 0.045) | 0.014 (-0.020, 0.049) | 0.039 (-0.012, 0.090)* |
| Past or current drinker^4^ | 0.024 (-0.016, 0.064) | 0.022 (-0.019, 0.064) | 0.032 (-0.017, 0.063) | 0.024 (-0.035, 0.082) |
| Pesticide exposure | -0.009 (-0.031, -0.013) | -0.004 (-0.027, 0.018) | -0.007 (-0.028, 0.015) | -0.013 (-0.045, 0.018) |
| Fruit (servings/week) | -0.001 (-0.001, 0.0004) | -0.00004 (-0.001, 0.001) | -0.0003 (-0.001, -0.001) | -0.002 (-0.003, -0.0003)** |
| Vegetable servings/week | -0.001 (-0.001, 0.001) | -0.0005 (-0.002, 0.0006) | -0.0003 (-0.001, 0.001) | 0.0003 (-0.001, 0.002) |
| Vendor (meals/month) | 0.0001 (-0.0001, 0.0003) | 0.0001 (-0.0001, 0.0004) | 0.0001 (-0.0001, 0.0003) | 0.00004 (-0.0003, 0.0004) |
| Physically active | -0.004 (-0.024, 0.017) | -0.018 (-0.039, 0.004)* | -0.011 (-0.031, 0.009) | 0.020 (-0.009, 0.050)* |
| Sleeping (hours/week) | 0.001 (-0.001, 0.0.002) | 0.0002 (-0.001, 0.001) | 0.0004 (-0.001, 0.001) | 0.001 (-0.0003, 0.003)* |
| BMI (kg/m^2^) | 0.004 (0.001, 0.008)** | 0.006 (0.002, 0.009)*** | 0.005 (0.002, 0.009)*** | 0.002 (-0.003, 0.007) |
| Waist (cm) | 0.022 (0.008, 0.035)*** | 0.029 (0.016, 0.043)**** | 0.026 (0.013, 0.039)**** | 0.009 (-0.011, 0.028) |
| Hip (cm) | 0.023 (0.010, 0.037)*** | 0.031 (0.017, 0.045)**** | 0.027 (0.014, 0.040)**** | 0.012 (-0.007, 0.032) |
| Visceral fat (cm) | 0.014 (0.006, 0.023)*** | 0.019 (0.010, 0.028)**** | 0.017 (0.008, 0.026)**** | 0.007 (-0.006, 0.019) |
| Subcutaneous fat (cm) | 0.075 (0.047, 0.102)**** | 0.099 (0.071, 0.127)**** | 0.088 (0.061, 0.115)**** | 0.035 (-0.006, 0.075)* |
| HDL-C (mmol/l) | 0.051 (0.019, 0.082)*** | 0.053 (0.024, 0.082)**** | 0.058 (0.033, 0.082)**** | 0.070 (0.033, 0.106)**** |
| LDL-C (mmol/l) | 0.019 (0.006, 0.032)*** | 0.018 (0.005, 0.031)*** | 0.018 (0.006, 0.031)*** | 0.022 (0.003, 0.040)** |
| TC (mmol/l) | 0.022 (0.010, 0.033)**** | 0.021 (0.009, 0.033)**** | 0.021 (0.010, 0.033)**** | 0.023 (0.006, 0.039)*** |
| TG (mmol/l) | 0.013 (-0.006, 0.033)* | 0.022 (0.002, 0.042)** | 0.018 (-0.001, 0.038)* | -0.004 (-0.032, 0.024) |

All blood pressure indices were log-transformed; 1education was coded as some formal education vs. no education; 2SES was coded as those with highest vs. those with lowest SES; 3smoking status was coded as those who are current or past smokers vs. those who never smoked; 4alcohol intake was coded as those who had ever drunk alcohol vs. those who had never drunk; *p value<0.2; **p value<0.05; ***p value<0.01; ****p value<0.001
